# Reducing sampling error in faecal egg counts from black rhinoceros (*Diceros bicornis*)^☆^

**DOI:** 10.1016/j.ijppaw.2013.10.002

**Published:** 2013-10-22

**Authors:** Andrew P. Stringer, Diane Smith, Graham I.H. Kerley, Wayne L. Linklater

**Affiliations:** a Centre for Biodiversity and Restoration Ecology, Victoria University of Wellington, New Zealand; b Centre for African Conservation Ecology, Nelson Mandela Metropolitan University, Port Elizabeth, South Africa

**Keywords:** Faecal egg counts, Parasitology, Sampling error, Black rhinoceros

## Abstract

Faecal egg counts (FECs) are commonly used for the non-invasive assessment of parasite load within hosts. Sources of error, however, have been identified in laboratory techniques and sample storage. Here we focus on sampling error. We test whether a delay in sample collection can affect FECs, and estimate the number of samples needed to reliably assess mean parasite abundance within a host population. Two commonly found parasite eggs in black rhinoceros (*Diceros bicornis*) dung, strongyle-type nematodes and *Anoplocephala gigantea*, were used. We find that collection of dung from the centre of faecal boluses up to six hours after defecation does not affect FECs. More than nine samples were needed to greatly improve confidence intervals of the estimated mean parasite abundance within a host population. These results should improve the cost-effectiveness and efficiency of sampling regimes, and support the usefulness of FECs when used for the non-invasive assessment of parasite abundance in black rhinoceros populations.

## Highlights

• Faecal egg counts should be taken from the centre of black rhinoceros dung.
• These are reliable up to 6 h after defecation.
• More than nine samples are needed per black rhinoceros population.

## Graphical abstract

**Figure:**
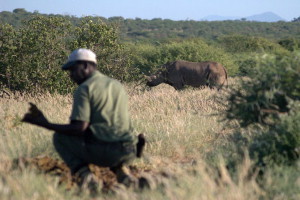

## 1 Introduction

Evaluating intestinal helminth infections is important for animal production and animal welfare. Parasites may impact population growth (Irvine, 2006) and so the abundance of parasites within populations and across meta-populations is important for the conservation of threatened species. Parasites are usually aggregated within a small proportion of a host population (Poulin, 2007). Gasbarre et al. (1996) concludes that greater than 15 samples are needed to ensure that 95% of the time the level of parasite aggregation is accurately estimated. Thus, large sample sizes are often needed to accurately capture the level of parasite abundance within a population.

In conservation management, culling an animal to determine its parasite intensity is rarely an option. Opportunistic autopsies on natural deaths can be useful but may give a biased sample of a population’s parasite abundance. For some intestinal parasites, faecal egg counts (FECs) are used as a non-invasive tool to determine the abundance of parasites within a host. The technique is particularly suitable for conservation mangers because it allows extensive and intensive non-invasive sampling of individual hosts and populations of hosts. Nevertheless, the technique does have constraints.

A direct and positive relationship between FECs and parasite burdens has been shown in numerous studies (Daş et al., 2011; McKenna, 1981; Rieu et al., 2007; Roberts and Swan, 1981; Seivwright et al., 2004; Sinniah, 1982; Sinniah et al., 1981; Sithithaworn et al., 1991). However an often cited concern with FECs is that this relationship may not be linear (Gillespie, 2006; Gooderham and Schulte-Hostedde, 2011). For instance, at lower parasite densities a sex-ratio bias towards female parasites may increase FECs independently of actual parasite population sizes (Poulin, 1997). Conversely, at high parasite densities, parasite ovulation rate may be reduced due to inter-specific competition (Christensen et al., 1995; Roepstorff et al., 1996; Sithithaworn et al., 1991). These patterns of variation must be considered during the interpretation of FECs but are not a source of methodological error.

It is recommended that FECs should only be used when samples have been taken rectally or directly after observed defecation (Zajac and Conboy, 2006). This is because temperature, light levels and oxygen availability may all be cues for the hatching of directly-transmitted parasites post-defecation (Nielsen et al., 2010). Furthermore the moisture content of faeces may change rapidly or eggs may be eaten by predators, thereby affecting the eggs per gram of faeces estimate (Anderson and Schad, 1985; Eberhard et al., 2001). These mechanisms may also differentially influence the FEC within the dung bolus, such as in the surface layer of dung in comparison to the centre, as the surface layer of dung is more exposed to the environment (Daş et al., 2011).

FECs are frequently used to evaluate the abundance of parasites within a host population. However, they are rarely used with elusive host species of conservation concern such as the black rhinoceros (*Diceros bicornis*). Known sources of methodological error come from laboratory techniques (Cringoli et al., 2004) and sample storage strategies (Dacombe et al., 2007; Seivwright et al., 2004; Zajac and Conboy, 2006). This paper will focus on potential sources of sampling error, primarily the collection of dung where defecation has not been observed. This paper investigates how sampling at known intervals following defecation, and from different locations within the faecal bolus, affects FECs from black rhinoceros. We also determine the minimum number of samples needed to accurately estimate mean parasite abundance within a population.

## 2 Materials and methods

FECs were performed on black rhinoceros (*Diceros bicornis*) dung. Two types of parasite eggs were commonly found, strongyle-type eggs (family Strongylidae), and a cestode, *Anoplocephala* sp. There are seven species of strongyle that infect black rhino in South Africa (*Kiluluma* spp. and *Khalilia rhinocerotis*), and only one cestode, *Anoplocephala gigantea* (Penzhorn et al., 1994; Knapp et al., 1997).

To test for differences between the centre and the surface layer of faecal boluses, 43 fresh boluses were sampled over four two-day sampling periods spread between Apr–Sep in 2011 from a wildlife reserve in the Eastern Cape of South Africa. Fresh faeces were collected during the early morning (dawn-10am) from latrines located along roads. To reduce the possibility of pseudoreplication a stratified random sampling regime, modified to ensure a minimum of 1 km between sample sites, was used. Black rhinoceros boluses are 12–15 cm in diameter. A sample of approximately 10 g of dung was collected from the centre of one complete bolus per dung pile. From the same bolus, another sample of approximately 10 g of dung was taken from the surface to a maximum depth of 1 cm. Each sample was stored at 4 °C in a sealed plastic bag, with excess air removed, until analysis (within 4–28 h) (Nielsen et al., 2010). A modified McMaster technique (Zajac and Conboy, 2006) using Sheather’s sugar solution was used for the flotation and enumeration of parasite eggs. Four replicate chambers were counted for each sample resulting in an analytical sensitivity of 25 eggs per gram (epg) of faeces per individual. Paired t-tests were used to compare differences between the surface layer and centre of each bolus sampled. The differences between these samples were normally distributed. SPSS (IBM, 2011) was used for all calculations unless otherwise stated.

To test how FECs may change due to a delay in sample collection, freshly deposited boluses (*n* = 7) were collected from black rhino captured for translocation and reintroduction, and held temporarily in purpose-built enclosures in the same Eastern Cape reserve. Boluses of dung were collected at dawn before the animals’ enclosures were cleaned. Boluses were judged freshly defecated if they were still warm. Boluses were placed outside the enclosures and subjected to normal daytime conditions. Approximately 10 g samples were taken from the centre of each immediately and then at 3 h intervals up until 9 h after initial collection. Boluses were reformed after each sampling event. Samples were stored and analysed as previously indicated. Strongyle egg maturity was estimated based on the internal structure of each egg. Morulated eggs (Zajac and Conboy, 2006) or those with no clear internal structure were classified as immature, while any egg where larvae or a pre-larval shape could be identified inside the egg were classified as mature. With time, strongyle eggs were expected to hatch and not be recoverable using the McMaster technique while *A. gigantea* eggs were not expected to hatch. Data was normally distributed and Mauchly’s sphericity test showed that sphericity could be assumed. We used a repeated measures ANOVA to test whether FECs and the level of egg maturity changed with time after defecation.

We then investigated how sample size affects the reliability of estimates of mean FEC for a population. Fresh faecal samples were collected from 18 populations of black rhinoceros from across eastern South Africa (Stringer, unpublished data), where parasite abundance, aggregation and sample size vary. Bootstrap 90% confidence interval estimations for the mean FEC using 2000 replications were calculated using the software “Quantitative Parasitology” (Rózsa et al., 2000) for each population. For each population, the size of these confidence intervals (as percentage distances from the mean) were then plotted against sample size. A multiple regression was then used to test how parasite aggregation, estimated mean abundance, and sample size affected confidence intervals. The level of parasite aggregation within a population was calculated using the corrected moment estimation of *k* which reduces bias caused by small sample size (Gregory and Woolhouse, 1993).

Five wildlife reserves from the previously mentioned dataset with sample sizes larger than nine were used to further investigate the precision of estimates of mean parasite abundance. First, data points were randomised. Then, after each sampling event, mean parasite abundance was calculated. The percentage distance of this mean from the best estimate of the mean using all data points was then calculated.

## 3 Results

FECs from the centre of faecal boluses were significantly higher than from the surface layer of boluses for both parasite groups when all four sampling periods were combined (strongyles: *T*_42_ = 6.65, *p* < 0.001. *A. gigantea*: *T*_42_ = 3.23, *p* = 0.002). Analysing the data for each sampling period revealed significant differences on three out of four sampling periods for strongyles and two out of four sampling periods for *A. gigantea*.

**Fig. 1:**
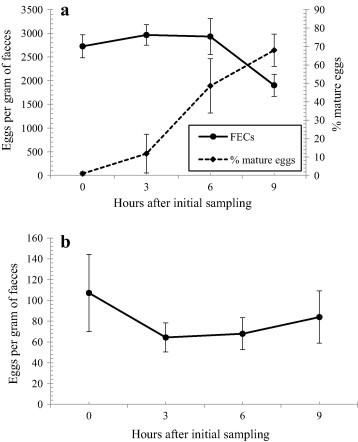
(a) Mean strongyle FECs and mean percentage of mature eggs, sampled 3, 6 & 9 h after initial collection from captive black rhino. Error bars represent ± 1 S.E. (b) Mean *A. gigantea* FECs, sampled 3, 6 & 9 h after initial collection from captive black rhino. Error bars represent ± 1 S.E.

For the time-specific FEC sampling, there was a significant reduction in strongyle FECs over time (RM ANOVA, *n* = 7, *F*_3,18_ = 5.1, *p* = 0.01), while the percentage of mature eggs increased through time (*F*_3,18_ = 15.1, *p* < 0.001). FECs did not decline until after 6 h had passed since defecation and initial collection (see Fig. 1). In a pairwise comparison of time points using a Bonferroni correction, the largest difference was between the 3 and 9 h collection points. *A. gigantea* FECs were slightly more variable, and did not change significantly through time (*F*_3,18_ = 0.07, *p* = 0.86).

**Fig. 2:**
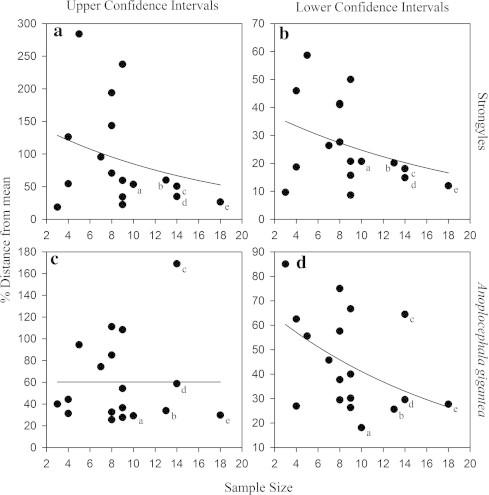
The distance from the mean of bootstrap 90% confidence intervals plotted against sample size for FECs for 18 black rhino populations. Lines represent the fitted negative exponential curve. Five populations are labelled “a–e” that are used in Fig. 3. (a) Strongyle upper confidence intervals. (b) Strongyle lower confidence intervals. (c) *A. gigantea* upper confidence intervals. (d) *A. gigantea* lower confidence intervals.

Lower confidence intervals for mean abundance improved as sample size increased for both parasite groups (see Fig. 2). Similarly, upper confidence intervals improved with increasing sample size for strongyles, but not for *A. gigantea* (although this trend appears to be driven by a single extreme value). Upper confidence limits were generally much further from the mean. Generally, confidence intervals were much improved when sample size was greater than nine (see Fig. 2).

Further investigation included the impacts of aggregation and calculated mean on the size of confidence intervals. A multiple regression revealed that the size of confidence intervals for strongyles was significantly predicted by parasite aggregation (Beta = −0.57, *p* < 0.01) and mean abundance (Beta = −0.61, *p* < 0.01) but not sample size (Beta = −0.11, n.s). The overall mode fit was *r*^2^ (adj) = 0.63. While, for *A.gigantea* confidence intervals were significantly predicted by parasite aggregation (Beta = −0.82, *p* < 0.001) but not mean abundance (Beta = −0.04, n.s) or sample size (Beta = −0.21, n.s). The overall model fit was *r*^2^ (adj) = 0.61.

**Fig. 3:**
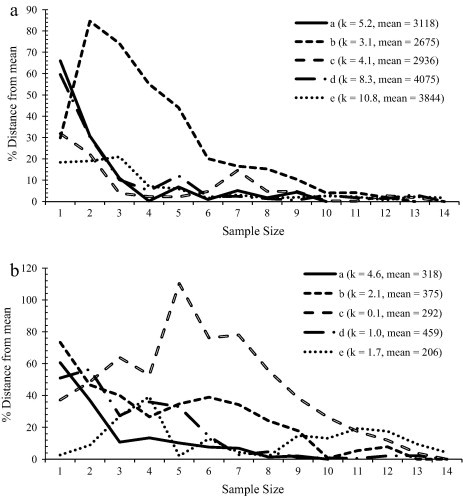
The accuracy of the estimated mean after each sampling event is plotted for each reserve with 10 or more samples. The corrected moment estimate of *k* and the population mean are given in the legend. (a) Strongyle (b) *A. gigantea.*

Using only those reserves with nine or more samples, after six samples, all estimates of mean parasite abundance were within 20% of the best estimate for strongyles (see Fig. 3a). The population that took longest to improve had the lowest mean abundance. While for *A. gigantea*, after nine samples 4 out of 5 reserves’ estimates of the mean were within 20% of the best estimate. The single reserve that took longer to improve (in Fig. 3b) is the same extreme result seen in Fig. 2c, and is associated with a high degree of parasite aggregation within that population.

## 4 Discussion

Parasite enumeration in free-ranging wildlife is often difficult and time consuming. There are many potential sources of error that may affect FECs. Identifying these sources of error is important so that they can be controlled by experimental design or considered during the interpretation of results.

Overall FECs were lower in faeces collected from the surface layer of boluses in comparison to the centre of bolus, although this trend differed between sampling periods. It is possible that this variation is caused by exposure to environmental conditions and predation, depleting egg density in the surface layer. Sampling from the centre of boluses may be a useful technique for host species with similar sized, or larger boluses, such as white rhino (*Ceratotherium simum*) and African elephant (*Loxodonta* spp.). In other host ungulate species, with smaller faecal boluses and larger surface area to volume ratios, environmental influences may more quickly penetrate the centre of boluses.

FECs were robust to quite long periods between defecation and sample collection. Strongyle eggs matured during the initial 6 hour period after defecation but this did not affect FECs. *A. gigantea* FECs did not decline, however these eggs do not develop into larvae as they are instead eaten by their intermediate host. Hence, the number of eggs eaten by intermediate hosts or predators, or decaying up until 9 hours after defecation was minimal. It must be noted that all FECs were calculated by the wet weight of the sample rather than the dry weight. Hence, it would be expected that as water evaporated from the dung calculated FECs would increase. We did not observe any increase in FECs indicating that actual egg numbers may have reduced over time or that evaporation from the centre of dung boluses was minimal.

A sampling regime that collects samples without observing defecation is only useful if faeces can be identified as being collected within 6 hours of defecation. Here, although the rate at which strongyle eggs mature was variable between samples, the level of egg maturation within the sample could be used to broadly assess the age of faeces. There could be other problems with collecting older samples. For instance, mature eggs may be more difficult to identify (Zajac and Conboy, 2006), although this was not found for the parasite groups studied here. Finally, if defecation is not observed, then accidental collection from the same individual would be possible. A stratified random sampling regime would reduce the chance of this pseudoreplication (Dytham, 2003), but not eliminate it.

Collecting more than nine samples greatly improved confidence intervals for the population mean. However, there was a great deal of statistical noise associated with this relationship. Some of this could be explained by the estimated mean – a smaller mean was associated with larger confidence intervals because our methodology had a resolution of 25 epg. Hence, if egg counts are low then the methodological resolution of egg counts should be increased. This may be done by decreasing the dilution factor or increasing the number of replicates from each sample (Torgerson et al., 2012).

A large amount of variation in the size of confidence intervals was explained by the level of parasite aggregation within a population. Hence, if parasite aggregation is high more samples may be required in order to improve confidence intervals. This reflects one of the disadvantages of reporting mean abundance in parasite studies – the mean is dependent on a few heavily infected individuals and is not accurately indicative of the typical infection across the population of hosts (Rózsa et al., 2000). This was shown in *A. gigantea* (Fig. 3b) where, although the accuracy of estimates of mean parasite abundance was positively associated with sample size, certain reserves required many more samples for mean abundance estimates to become accurate. This was caused by a few, heavily parasitized individuals.

Depending on the study aims, the mean level of infection need not be determined at all. For instance, Generalised Linear Mixed Models allow for non-normally distributed data and random effects, hence mitigating the need to reduce each population to a single data point (Bolker et al., 2009). The required sample size is then dependent on the study questions – whether it is the typical level of infection or the heavily parasitized individuals that are of interest.

These results should reduce the costs and labour of data collection, and increase the usefulness of FECs as a tool for the non-invasive assessment of parasite abundance. Although this study was specific to black rhino, the techniques used here could apply to numerous other host species. However, studies wishing to use a delayed faecal sampling strategy must independently test the speed at which FECs decline. Quick and easy methods of parasite enumeration will assist conservation managers identify when parasites may be of concern for the conservation of threatened species.
